# A graph exploration method for identifying influential spreaders in complex networks

**DOI:** 10.1007/s41109-017-0047-y

**Published:** 2017-08-14

**Authors:** Nikos Salamanos, Elli Voudigari, Emmanuel J. Yannakoudakis

**Affiliations:** Department of Informatics, Athens University of Economics and Business, 76, Patission Street, Athens, 10434 Greece

**Keywords:** Influential spreaders, Complex networks, Graph mining

## Abstract

The problem of identifying the influential spreaders - the important nodes - in a real world network is of high importance due to its theoretical interest as well as its practical applications, such as the acceleration of information diffusion, the control of the spread of a disease and the improvement of the resilience of networks to external attacks. In this paper, we propose a graph exploration sampling method that accurately identifies the influential spreaders in a complex network, without any prior knowledge of the original graph, apart from the collected samples/subgraphs. The method explores the graph, following a deterministic selection rule and outputs a graph sample - the set of edges that have been crossed. The proposed method is based on a version of *Rank Degree* graph sampling algorithm. We conduct extensive experiments in eight real world networks by simulating the *susceptible-infected-recovered (SIR)* and *susceptible-infected-susceptible (SIS)* epidemic models which serve as ground truth identifiers of nodes spreading efficiency. Experimentally, we show that by exploring only the 20% of the network and using the degree centrality as well as the k-core measure, we are able to identify the influential spreaders with at least the same accuracy as in the full information case, namely, the case where we have access to the original graph and in that graph, we compute the centrality measures. Finally and more importantly, we present strong evidence that the degree centrality - the degree of nodes in the collected samples - is almost as accurate as the k-core values obtained from the original graph.

## Introduction

Understanding spreading process in real world complex networks is a central subject in network analysis, due to the variety of applications which occur - such as the control of the spread of a disease, the viral marketing, as well as the network vulnerability to external attacks. Key role in these processes play the high spreading efficient nodes which are often called *influential spreaders*, representing the nodes that are more likely to spread information or a virus in a large part of the network.

Thorough research has been realized in order to connect the topological properties of network nodes with their spreading efficiency. Kitsak et al. (2010) proposed the k-core decomposition method (Seidman 1983) as an *influential spreaders identifier*, showing that the k-core values constitute a more reliable measure than *degree centrality* and *betweenness centrality*. One of the core results is that the placement of a node (node global property) is more important than its degree (node local property). That is, two nodes with the same degree but different placement in the network, where the one is connected with the periphery of the network and the other one with the innermost core may not have equal spreading efficiency. Thus, highly connected nodes are not always the best spreaders, while less connected nodes but, at the same time, well connected with the core of the network may strongly affect the spreading process.

In addition, Zeng and Zhang (2013) investigated the limitations of the k-core method and proposed a mixed degree decomposition procedure which performs more efficiently than the k-core approach. Chen et al. (2012) proposed the *local centrality*, a semi-local centrality measure, as a tradeoff between the degree centrality (local measure) and the computationally complex betweenness centrality and closeness centrality (the global measures). They showed that local centrality is more effective to identifying influential nodes than the degree centrality.

Hébert-Dufresne et al. (2013) performed a very large study of local and global centrality measures, along with a semi-local one which is based on the notion of community-structure, that is the number of communities that the nodes belong to. In order to define this overlapping community structure, the authors used the *Jaccard coefficient* measure - two links from a given node belong to the same community if their Jaccard coefficient is above a given threshold. Then, based on this community-structure approach, they introduced the notion of *structural hubs*, that is the nodes that connect a large number of communities.

Furthermore, several algorithms have been proposed such as the *LeaderRank* (Lü et al. 2011), a ranking algorithm for identifying influential nodes in directed social networks. LeaderRank is a parameter-free random walk algorithm analogous to PageRank (Brin and Page 1998). Moreover, Li et al. (2014) proposed a weighted variation of Leader Rank which outperforms LeaderRank. Furthermore, in Chen et al. (2013), the authors introduced *ClusterRank* - a local ranking algorithm for directed graphs that takes into account the nodes *clustering coefficient* and proved that ClusterRank outperforms other approaches such as LeaderRank.

In this paper we deal with the problem of identifying the influential spreaders of a complex network when we are not able to analyze the whole network directly, either because of its large size or of our limited computational resources which are necessary for estimating global centrality measures or other advanced nodes properties. Our approach is based on graph sampling - the problem of selecting a small subgraph which will preserve the topological properties of the original graph.

A first preliminary study which investigates the applications of graph sampling to the influential spreaders identification problem has been conducted in Salamanos et al. (2016), where we studied the effectiveness of *Rank Degree* as *influential spreaders* identifier. The Rank Degree is a graph exploration sampling method which can produce representative samples/subgraphs from an unknown graph, using only local information, that is the degree of the visited nodes (Voudigari et al. 2016; Salamanos et al. 2017).

In this paper, we extend the work in Salamanos et al. (2016), on several levels. First, we devise the *susceptible-infected-recovered (SIR)* and *susceptible-infected-susceptible (SIS)* epidemic models - a common approach in the literature - in order to define a kind of “ground truth” ranking of the graph nodes with regard to their spreading efficiency. Secondly, we perform a larger scale simulation of the sampling algorithms, in a larger collection of datasets. Finally, we study three well known sampling methods and we experimentally show that our method significantly outperforms all of them.

The experimental results for eight real world networks demonstrate that by exploring only 20% of the network and using the degree centrality, the k-core, as well as the betweenness centrality, we are able to identify the influential spreaders with at least the same accuracy as the accuracy achieved in the *full information* case. This case is the one by which we have access to the original graph and in that graph we compute the centrality measures. Furthermore, we present strong evidence that the degree centrality - the degree of nodes in the collected samples - is almost as accurate as the k-core measure computed in the original graph.

## The rank degree method

The approach we have followed in this paper is based on the Rank Degree sampling algorithm, which outperforms several other well known approaches. We concentrate our analysis in one of the Rank Degree versions, which for the rest of the paper we call as maxRD. The maxRD can be summarized as follows:

Given a graph *G*(*N,E*) do the following:

1. Start *s* parallel graph traverses from *s* randomly chosen nodes.
2. For each visited node *i* do:
3. Select his max-degree friend *j*.
4. Visit *j*.
5. Repeat, without crossing the same edges for a second time.
6. Halt, when the number of discovered nodes has reached a given target size *x*.
7. Output the discovered subgraph - the set of edges that have been crossed.

Algorithm 1 presents the maxRD in details. The main characteristic of this method is that the graph traverse is based on a deterministic selection rule (Step 10) - the ranking of nodes according to their degree values (local information). The only parameter of the algorithm is the number *s* of the initial starting nodes (seeds). The algorithm, starting from *s* initial nodes, performs *s* parallel graph traverses without crossing the same edges for a second time. The visited nodes remain present in the graph and only the selected edges are removed (Step 14). Thus, any selected node can be visited many times, but not from the same paths. This edge-elimination process alters the original graph and eventually the nodes degree and ranking. Hence, neither the nodes degree nor the graph are stable.


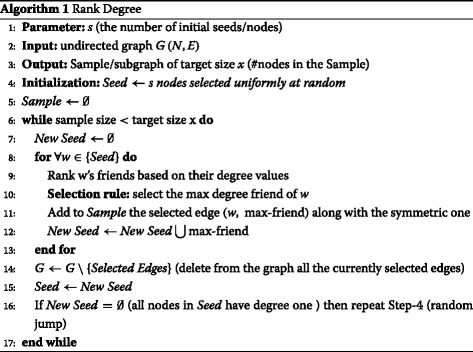


A detailed analysis of the algorithm can be found in Voudigari et al. (2016) and Salamanos et al. (2017), where we have thoroughly studied the properties and the efficiency of the algorithm as well as other variations of the selection rule.

As we previously mentioned, the main phase of the algorithm is deterministic, but in the extreme case, where all the current seeds have degree equal to one, the algorithm executes a random jump (Step 16) in order not to get trapped.

As we shall see in the next sections, this simple exploration algorithm can effectively identify the influential spreaders in a complex network, by using as a measure the degree of nodes in the collected subgraphs.

## Methods

### Sampling methods

Apart from our method, we study the *Forest Fire*, *Metropolis Hastings Random Walk* and *Metropolis-Hastings*, three well known sampling methods in the literature. The first two methods are graph exploration algorithms, while the *Metropolis Hastings* is a “centralized” algorithm, which takes as input the original graph.

**Forest Fire (FF)** (Leskovec et al. 2005; Leskovec and Faloutsos 2006; Leskovec et al. 2007) algorithm starts from a randomly selected node (seed); at each step, the algorithm moves from the current set of seeds to the next one, as follows: from each node *w* in the set of current nodes (seeds), a random number *x* is generated which is geometrically distributed with mean *p*_*f*_(1−*p*_*f*_). Then, *x* outgoing edges are selected randomly from the set of node *w* outgoing edges. The end nodes of the selected edges constitute the next set of current nodes (seeds). At each step, the visited nodes are considered as burned and are removed from the graph. Hence, they cannot be traversed for a second time. Finally, the process is repeated until a sample of the requested size is reached. The parameter *p*_*f*_ is called *forward burning probability*. According to Leskovec and Faloutsos (2006), best performance is obtained for *p*_*f*_≥0.6. In our experiments, we set *p*_*f*_=0.7.

**Metropolis-Hastings Random Walk (MHRW)** (Stutzbach et al. 2009; Gjoka et al. 2011; Li et al. 2015) is an application of the Metropolis algorithm (Metropolis et al. 1953), for uniform sampling. It modifies the Random Walk algorithm, as follows: (i) First, select a node *x*, uniformly at random. (ii) Select a neighbor *y* of *x*, uniformly at random. (iii) Find the degree of *y*. (iv) Generate a random number *p*, uniformly between 0 and 1. If $p \leq \frac {degree(x)}{degree(y)}$, *y* is the next step. Otherwise, remain at *x*, as the next step.

**Metropolis-Hastings (MH)** (Hübler et al. 2008) is a sampling algorithm that is able to collect representative graph samples of small size. Given a target sample size *n*, the algorithm initially selects *n* nodes, at random, the edges of which form an initial graph sample. Then, by incorporating the Metropolis algorithm, together with some predefined graph properties, the algorithm adds and removes nodes from the current sample, until the graph properties in question are optimized. Hübler et al. (2008) proposed several sampling strategies; one of the best uses the degree distribution as the optimized property. This is the version of Metropolis-Hastings, implemented in this paper.

### Epidemic models

In the absence of ground truth information, as regards the nodes spreading efficiency, several approaches have been proposed in the literature, such as the basic epidemic models, as well as the *linear threshold* and *independent cascade* models (Kempe et al. 2003). In this paper, we devised the standard epidemic models *susceptible-infected-recovered (SIR)* and *susceptible-infected-susceptible (SIS)*, which tend to simulate the spreading process in a graph (Kitsak et al. 2010; Chen et al. 2012; Hébert-Dufresne et al. 2013).

#### The SIR model

In the SIR model, the individuals/nodes can appear in three states - susceptible (S), infected (I) and recovered (R). Each infected node can transmit the disease to any susceptible node which is connected with, with probability *β* per unit time (infection rate) and at the same time, it can recover from the disease and become immune, with probability *γ* per unit time (recovery rate).

The dynamics of SIR model exhibits a *phase transition* where the control parameter of the process is the ratio *T*=*β*/*γ*. A critical point exists, the *epidemic threshold* $T^{c} = \frac {\langle k \rangle }{\langle k^{2} \rangle - \langle k \rangle }$, beyond which an epidemic outbreak ensues. The 〈*k*〉 and 〈*k* ^2^〉 are the first and second moments of the degree distribution *P*(*k*) and in our case, they correspond to the average degree and the average squared degree, respectively. For *T*<*T* ^*c*^ only a limited number of individuals are infected, whereas for *T*≥*T* ^*c*^ (epidemic phase) an epidemic outbreak occurs and the disease infects a finite fraction of the population - the total size of the outbreak - which corresponds to a large connected component (Castellano and Pastor-Satorras 2010; Newman 2010; Pastor-Satorras et al. 2015). Finally, there is a mapping between the SIR model and the *bond percolation*, as proved in Newman (2002).

In our experiments, without lack of generality, we set *γ*=1. Moreover, we assume that one node is initially infected and all the other nodes are susceptible to the disease.

#### The SIS model

In the SIS model, the nodes appear in two states, susceptible (S) and infected (I). Each infected node can infect any susceptible node which is connected with, with probability *ν* per unit time (infection rate) and with probability *δ* per unit time (recovery rate) it can become healthy again - but at the same time, susceptible to future infection- defining the effective spreading rate *λ*=*ν*/*δ*.

In the case of the SIS dynamics, a *phase transition* occurs at the critical point (epidemic threshold) $ \lambda ^{c} = \frac {\langle k \rangle }{\langle k^{2} \rangle }$ of the control parameter *λ*; the 〈*k*〉 and 〈*k* ^2^〉 have been described previously. When *λ*<*λ* ^*c*^, the disease eventually vanishes from the graph, whereas for *λ*≥*λ* ^*c*^ (epidemic phase) there is a dynamically stable equilibrium state, where the average density of infected nodes is stable - in other words, equal amount of infections and recoveries (Pastor-Satorras and Vespignani 2001; Castellano and Pastor-Satorras 2010; Newman 2010; Pastor-Satorras et al. 2015).

In the simulations, without lack of generality, we set *δ*=0.8. We also set that the 20% of the individuals/nodes are initially infected.

### Evaluation measures

#### Top-k spreading efficiency

In the SIR epidemic, the spreading efficiency of a given node *i* is defined by the size of the population *M*_*i*_ that will eventually get infected when the epidemic is originated at the node *i*. Hence, the ground truth ranking, that is referring to the SIR model, is produced by the *M*_*i*_ values.

The *imprecision function* Kitsak et al. (2010) is given by:

1 $$ \epsilon(\text{top-k}) = 1- \frac{\overline{M}_{C}(\text{top-k})}{\overline{M}_{eff}\text{(top-k)}}  $$

where $\overline {M}_{C}(\text {top-k})$ is the $\frac {\sum _{i \in N_{\text {top-k}}} M_{i}}{|N_{\text {top-k}}|}$, i.e. the average *M*_*i*_ values of the top-k nodes, when the ranking is based on the nodes centrality values *C*. The *N*_top-k_ is the set of nodes in the top-k. The $\overline {M}_{eff}\text {(top-k)}$ is the average *M*_*i*_ values of the top-k nodes, in the ground truth ranking.

In the SIS epidemic, we define the *persistence-distance*, based on the notion of *persistence*, (Kitsak et al. 2010), namely the probability *ρ*_*i*_(*t*) that a given node *i* is infected at time *t*. At the equilibrium state, the probability *ρ*_*i*_(*t*→*∞*) is independent of *t* (Pastor-Satorras and Vespignani 2001). The persistence is a measure of the importance of a given node during the epidemic process; it represents the frequency that the node in question has been infected by the disease. If we consider the SIS model as a rumor spreading process in a social network, then the persistence corresponds to the probability that a given individual gets informed about a certain rumor and consequently, he is able to pass this rumor further into the network.

We define the ground truth ranking in SIS with reference to the persistence values. Hence, the *persistence-distance* is defined as:

2 $$ \delta \rho(\text{top-k}) = 1- \frac{\overline{\rho}_{C}(\text{top-k})}{\overline{\rho}_{eff}(\text{top-k})}  $$

where $\overline {\rho }_{C}(\text {top-k})$ is the average persistence of the nodes in the top-k, when the ranking is based on the centrality measure *C*. The $\overline {\rho }_{eff}(\text {top-k})$ is the average persistence of the nodes in the top-k, according to the ground truth ranking.

In this paper, we use three centrality measures, the *degree centrality*, the *k-core decomposition* and *betweenness centrality* - widely used for the influential spreaders identification problem (Kitsak et al. 2010; Hébert-Dufresne et al. 2013).

#### Top-k nodes similarity

We apply the *OSim* (Haveliwala 2003), an object similarity measure (in our case the objects are the nodes), which measures the common elements between two ranking lists *A* and *B* (each of size k), without taking into account their ordering. It is defined as $OSim(A,B)=\frac {|A \cap B|}{k}$. In our case, the lists *A* and *B* correspond to the top-k nodes in the ranking lists $r_{eff}^{G}$ and *r*_*C*_, thus, the OSim is given by:

3 $$ OSim(\text{top-k})=\frac{\left|r_{eff}^{G}(\text{top-k}) \cap r_{C}(\text{top-k})\right|}{k}  $$

where $r_{eff}^{G}(\text {top-k})$ is the top-k nodes of the ground truth ranking $r_{eff}^{G}$ in the original graph. It is based on the nodes spreading efficiency - a notion that we have previously described. The *r*_*C*_(top-k) is the top-k nodes of the subjective ranking *r*_*C*_, which is based on the values of a given centrality measure *C*. The *r*_*C*_ is referring to either the original graph or to the samples that are generated by a sampling algorithm. When we study the general effectiveness of a given centrality measure *C*, then we compute the $r_{C}^{G}({top-k})$, which is referring to the ranking of nodes based on their centrality values (in descending order), obtained from the original graph *G*. When we study the effectiveness of a given sampling algorithm, then we have the $r_{C}^{S}(\text {top-k})$, which is the ranking of nodes in terms of their centrality values obtained from the samples - the generated subgraphs by the sampling algorithm in question. We note that in the case of samples, the centrality measure is computed in the sample subgraph structure.

#### Ranking similarity

Given a sample subgraph *S* and a centrality measure *C*, we apply the *Kendall tau* (Kendall 1938), the well known rank correlation coefficient measure, which measures the relative ordering between all pairs in two ranking lists *A* and *B*, when *A* and *B* are consisting of the same elements. The *A* list is the $r_{C}^{S}(\text {top-k})$ that we mentioned previously in the OSim definition. The *B* list is the ranking list of the values that the top-k sample nodes have in the ground truth ranking $r_{eff}^{G}$, regardless of their position in the $r_{eff}^{G}$. We follow this approach because some of the nodes in the $r_{C}^{S}(\text {top-k})$ may not appear in the top-k of the ground truth ranking $r_{eff}^{G}$. Finally, we follow the same procedure when we study the effectiveness of the centrality measure based on the original graph (full information case). In this case, the A list corresponds to the $r_{C}^{G}({top-k})$, which has also been described previously.

### Datasets

We have used eight datasets of different types (social networks, collaboration networks, location based social network etc.), previously used for graph mining (Leskovec and Krevl 2014). We restrict our analysis to undirected graphs, therefore, we transform the directed graphs to undirected ones, by applying the symmetric one to each edge, after removing the self loops, if any.

- **D1** ***egoFacebook:*** undirected graph of 4039 users’ “friends-list”, i.e. the ego-net, from Facebook (McAuley and Leskovec 2012).
- **D2** ***wiki-Vote:*** voting network - directed graph - from *Wikipedia* consisting of 7115 users (Leskovec et al. 2010).
- **D3** ***CA-CondMat:*** scientific collaborations network - undirected - between 23,133 authors with paper submitted to Condense Matter category (Leskovec et al. 2007).
- **D4** ***p2p-Gnutella30:*** Gnutella *p*2*p* network topology - directed - of 36,682 nodes (hosts in the Gnutella network) (Ripeanu et al. 2002; Leskovec et al. 2007).
- **D5** ***Email-Enron:*** email communication network - undirected - of 36,692 nodes (email addresses) (Leskovec et al. 2009).
- **D6** ***loc-Brightkite:*** online location-based social network - undirected - of 58,228 nodes (Cho et al. 2011).
- **D7** ***soc-Epinions1:*** web of trust, obtained from Epinions - directed - of 75,879 nodes, members of www.epinions.com (Richardson et al. 2003).
- **D8** ***soc-Slashdot0922:*** social network - directed - of 82,168 nodes/users (Leskovec et al. 2009).

### Simulation setup

For each dataset, as well as, for each sampling method separately, we collect 100 samples, for 20% sample size. For maxRD, the number of initial seeds is defined as the 1% over the number of nodes in the original graph. The number of initial seeds is equal to one for the FF. A condition of the MHRW is that the graph is connected. In this paper, we study the real world graphs as they are - hence, they may not be well connected. In this case, it is possible that the MHRW will get trapped in a small region of the graph. In order to avoid that, we set the number of initial seeds equal to 1%. Furthermore, in each sampling trial, the iterations of MH are set to 20,000 and 30,000 for the datasets D1 - D5 and D6 - D8, respectively.

We study ten top-k intervals of 1*%*,2*%*,…,10*%*; namely, we study the first one percent, up to ten percent of the graph nodes. Hence, we compute the imprecision, persistence-distance, OSim and Kendall tau for each top-k, separately.

The simulation experiments have been implemented in MATLAB. The centrality values of the nodes in the samples, as well as in the original graphs have been computed using the igraph R package (Csardi and Nepusz 2006).

## Results

The overall steps of analysis that we have followed during this study can be summarized as follows:

Given a graph *G*(*N,E*), a centrality measure and a sampling algorithm:

1. **Ground-truth ranking:** Simulate SIR and SIS epidemics in *G* (1000 simulations per epidemic model). Rank the nodes based on their average epidemic efficiency (see “Methods” section).
2. **Samples-ranking:**
  - Run the sampling algorithm (100 samples/subgraphs, 20% sample size).
  - Compute the nodes centrality values in the samples subgraph structure.
  - Rank the nodes based on the nodes centrality values.
3. **Graph-ranking:** Full information case. Rank the nodes of *G* based on the nodes centrality values in *G*.
4. **Evaluation:** Find the imprecision and persistence-distance values, OSim and Kendall tau, for a given top-k, based on Samples and Graph rankings. Compare Samples-ranking accuracy vs Graph-ranking accuracy, in terms of the identified top-k influential spreaders.

### Estimating the epidemic parameters

A common approach in the literature is that the simulation of the epidemic models is performed at the largest connected component of a given network. In this paper, we study the real world networks as they are, hence other smaller components may exist. Thus, we cannot directly apply the theoretical epidemic threshold values. Furthermore, our final goal is to produce a ground truth ranking, by which we can study the top-1% to top-10% of the most influential nodes in the graph. For this ranking to be valuable, the values of *T*=*β*/*γ* and *λ*=*ν*/*δ* have to be above the epidemic threshold - but not very far from it. In all experiments, we set *γ*=1 and *δ*=0.8, as we mentioned previously in “Methods” section.

For both epidemic models, we set the parameter *p*=1,1.1,1.2,… and we express the *T*=*p*×*T* ^*c*^ and *λ*=*p*×*λ* ^*c*^. We simulate both SIR and SIS, for several *p* values, until a predefined criterion is roughly satisfied.

In each simulation instance of SIR model, we measure the epidemic efficiency of each node *i*, namely, the size of infected population in the long run, when the epidemic is initiated in *i*. Thus, each simulation instance is consisting of |*N*| trials, where |*N*| is the number of nodes. We repeat this process for 1000 independent simulations. Then, we select the smallest *p* value, per dataset, for which, the top-20% of the most effective nodes have, as population, average epidemic efficiency larger than the 1% of the graph, in every simulation instance. In Table 1, we present the *T* ^∗^=*p*×*T* ^*c*^, per dataset. These are the values that we have used for the rest of the analysis.

**Table 1:** Estimating the epidemic parameters

Dataset	D1	D2	D3	D4	D5	D6	D7	D8
〈*k*〉	43.69	28.32	8.08	4.82	10.02	7.35	10.69	12.27
〈*k* ^2^〉	4656.14	4117.03	178.20	55.18	1403.62	468.42	1966.47	1837.40
SIR								
*T* ^*c*^	0.0095	0.0069	0.0475	0.0956	0.0072	0.0159	0.0055	0.0067
*T* ^∗^	1.2×*T* ^*c*^	1.5×*T* ^*c*^	1.2×*T* ^*c*^	1.2×*T* ^*c*^	3×*T* ^*c*^	2.5×*T* ^*c*^	3×*T* ^*c*^	3×*T* ^*c*^
SIS								
*λ* ^*c*^	0.0094	0.0069	0.0453	0.0873	0.0071	0.01557	0.0054	0.0067
$\widehat {\lambda }$	*λ* ^*c*^	1.3×*λ* ^*c*^	*λ* ^*c*^	1.1×*λ* ^*c*^	1.5×*λ* ^*c*^	*λ* ^*c*^	1.2×*λ* ^*c*^	1.3×*λ* ^*c*^
*λ* ^∗^	*λ* ^*c*^	1.3×*λ* ^*c*^	*λ* ^*c*^	1.1×*λ* ^*c*^	2×*λ* ^*c*^	1.5×*λ* ^*c*^	2×*λ* ^*c*^	2×*λ* ^*c*^

*T* ^*c*^ and *λ* ^*c*^ are the theoretical epidemic thresholds for SIR and SIS, respectively. $\widehat {\lambda }$ are the lowest values for which an epidemic phase occurs, that is, the density of infected nodes is non-zero, in the long run. *T* ^∗^ and *λ* ^∗^ are the values used in the experiments (see for details the “Estimating the epidemic parameters” section)

In SIS model, in each simulation instance, the population of initially infected nodes is set to 20% of the nodes in the graph. We run the model, above the epidemic threshold until the dynamically stable equilibrium state has been reached, that is, the average density of the infected nodes is stable. We measure that by tracking the difference *Δ*(*I*)=*I*(*t*+1)−*I*(*t*), where *I*(*t*) is the density of infected nodes at time period *t*. In the first periods, the *Δ*(*I*) is always negative. When, for the first time we have *Δ*(*I*)≥0 then, from that point on, we leave the system to rest for 100 time periods. Then, from that point, we store the *Δ*(*I*)=*I*(*t*+1)−*I*(*t*) values. If for 100, or more, time periods, the average of the *Δ*(*I*) values is close to zero, then we assume that the equilibrium has been reached. In other words, during the last periods (more than 100), the overall gain or loss must be close to zero.

In Table 1, we present the smallest $\widehat {\lambda } = p \times \lambda ^{c}$, per dataset, for which *I*_*t*→*∞*_≠0, namely the density of infected nodes is non-zero, in the long run. In other words, for $\lambda \geq \widehat {\lambda }$ an epidemic phase occurs, that is, the disease does not vanish from the graph, instead a dynamically stable equilibrium emerges eventually. For instance, we observe in Table 1, that for the dataset D2 (*wiki-Vote*) we have $\widehat {\lambda } = 1.3 \times \lambda ^{c}$. This means that the theoretical *λ* ^*c*^ value does not correspond to the real epidemic threshold of the network, since for this value we do not observe a transition to the epidemic phase.

Finally, we estimate the smallest $\lambda ^{*} \geq \widehat {\lambda }$, for which the average density of the infected nodes at the equilibrium is at least 1% of the graph. We have used these *λ* ^∗^ values for the rest of the analysis.

### Top-k spreading efficiency

**Fig. 1:**
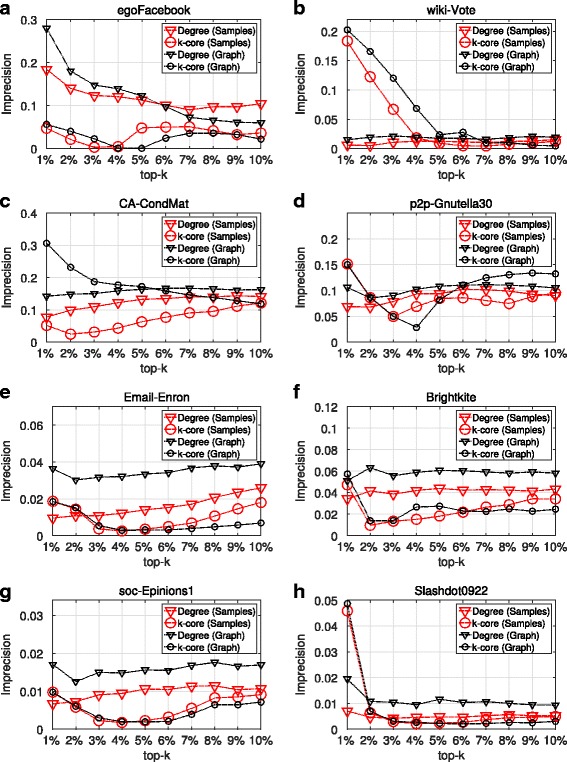
Top-k spreading efficiency, SIR epidemic. **a**-**h** Imprecision values in terms of degree centrality and k-core obtained from maxRD samples, as well as from the original graph

**Fig. 2:**
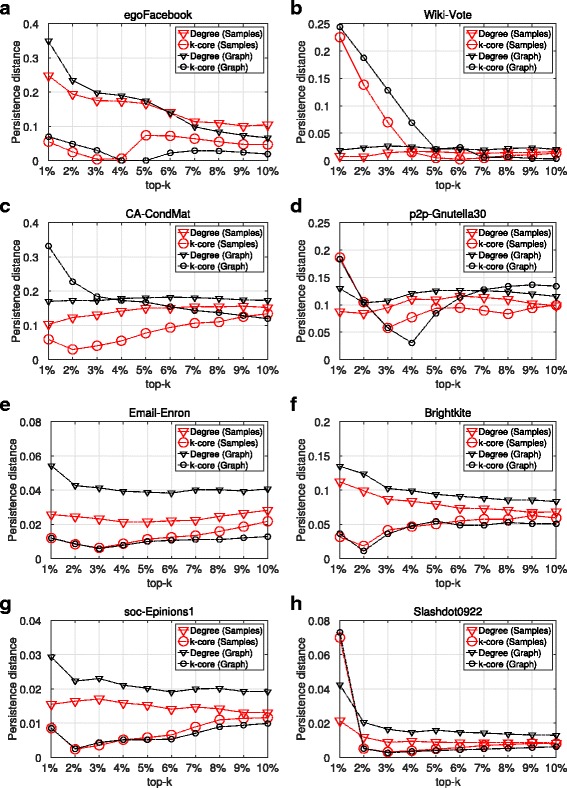
Top-k spreading efficiency, SIS epidemic. **a**-**h** Persistence-distances in terms of degree centrality and k-core obtained from maxRD samples, as well as from the original graph

Figures 1 and 2 as well as Table 2 present the results of SIR and SIS epidemics, with regard to the spreading efficiency of the top-k nodes - imprecision and persistence distances - as they have been identified by the maxRD samples. That is, we compute the nodes centrality values in the samples subgraph structure and then, using the Eqs. 1 and 2, we compute the imprecision and persistence distances per top-k. In addition to this, for the full information case, we present the imprecision and persistence distances for the same centrality measures, but this time, the centrality values are computed in the original graph *G*.

**Table 2:** Betweenness centrality, SIR and SIS epidemics

		Top-1%		Top-2%		Top-3%		Top-4%		Top-5%	
		maxRD	G	maxRD	G	maxRD	G	maxRD	G	maxRD	G
SIR	D1	**0.5857**	0.8198	**0.5380**	0.8295	**0.4914**	0.8231	**0.4182**	0.8231	**0.3656**	0.8216
	D2	**0.0391**	0.0990	**0.0807**	0.1092	**0.0792**	0.1092	**0.0549**	0.1101	**0.0460**	0.1279
	D3	**0.2506**	0.3575	**0.3040**	0.3836	**0.3112**	0.4029	**0.3135**	0.4016	**0.3022**	0.4118
	D4	**0.0668**	0.1230	**0.0699**	0.1046	**0.0822**	0.1052	**0.0933**	0.1107	**0.0994**	0.1095
	D5	**0.1499**	0.2394	**0.1797**	0.2739	**0.2159**	0.3035	**0.1789**	0.3127	**0.1505**	0.3134
	D6	**0.2037**	0.2089	**0.2350**	0.2606	**0.2401**	0.2756	**0.2675**	0.2822	**0.2381**	0.2833
	D7	**0.0963**	0.1109	0.1636	**0.1484**	0.1711	**0.1685**	0.2388	**0.1868**	**0.1771**	0.1890
	D8	**0.0312**	0.0485	**0.0346**	0.0453	0.0759	**0.0504**	**0.0538**	0.0625	**0.0418**	0.0739
SIS	D1	**0.6883**	0.8584	**0.6657**	0.8618	**0.6054**	0.8665	**0.5162**	0.8717	**0.4538**	0.8726
	D2	**0.0475**	0.1056	**0.0899**	0.1198	**0.0850**	0.1169	**0.0600**	0.1144	**0.0503**	0.1308
	D3	**0.2887**	0.4054	**0.3359**	0.4175	**0.3380**	0.4326	**0.3366**	0.4260	**0.3229**	0.4345
	D4	**0.0877**	0.1567	**0.0881**	0.1290	**0.0997**	0.1272	**0.1109**	0.1317	**0.1159**	0.1280
	D5	**0.1649**	0.2575	**0.1767**	0.2588	**0.2015**	0.2769	**0.1640**	0.2821	**0.1364**	0.2794
	D6	**0.3852**	0.3980	**0.3607**	0.3808	**0.3390**	0.3657	**0.3416**	0.3534	**0.3029**	0.3423
	D7	**0.1286**	0.1500	0.1697	**0.1609**	0.1686	**0.1676**	0.2201	**0.1771**	**0.1609**	0.1737
	D8	**0.0706**	0.0965	**0.0493**	0.0657	0.0769	**0.0589**	**0.0541**	0.0657	**0.0410**	0.0724

Imprecision (SIR epidemic) and persistence-distance (SIS epidemic) - in terms of betweenness centrality computed in maxRD samples, as well as, in the original graph *G*. The table entries contain the pairs (samples-value, graph-value), per top-k. Bold values are the lowest values per top-k and dataset, which according to the definitions in “Methods” section represent the best case. The maxRD prevails

First, in regard to the general effectiveness of the three centrality measures, when they are obtained from the original graph (full information case), we observe the following: In SIR, the accuracy of degree centrality and k-core are equivalent in two out of eight datasets (see Fig. 1 c, d), whereas, the k-core is clearly superior in five out of eight datasets (see Fig. 1 a, e, f, g, h). Similar is the outcome of SIS, where, in five datasets, the k-core outperforms degree centrality (see Fig. 2 a, e, f, g, h). Finally, comparing the values in Table 2 with reference to graph *G* and betweenness centrality with the corresponding values of degree centrality and k-core in Figs. 1 and 2, we conclude that the betweenness centrality has the lowest accuracy between the three centrality measures. Only in *p2p-Gnutella30* (D4), is the accuracy of betweenness centrality close to the one of degree centrality, in both epidemic models. These results partially verify the work of Kitsak et al. (2010), that k-core is superior both to degree centrality and betweeneess centrality.

With respect to maxRD efficiency, we observe that in both, SIR and SIS and for all eight datasets, the maxRD identifies the influential spreaders for all centrality measures, with equal or even higher accuracy than the one exhibited in the full information case.

Specifically, studying the plots in Fig. 1 (SIR epidemic), we observe that in any top-k, the imprecision values of maxRD samples are equal to or lower than the imprecision values for the same centrality measures computed in the original graph (i.e. the full information case). Moreover, in four out of eight datasets - *Email-Enron*, *Brightkite*, *soc-Epinions1* and *Slashdot0922* - the imprecision values, in terms of the degree centrality of the maxRD samples, are clearly lower than the imprecision values in terms of the degree centrality of the original graph (see Fig. 1 e, f, g, h).

The results for SIS epidemic are very similar with those of SIR. From Fig. 2, we conclude that using the maxRD samples, we are able to identify the influential spreaders with at least the same accuracy as if we had used the original graph. Moreover, in five out of eight datasets, the degree centrality of maxRD samples outperforms the degree centrality obtained from the original graph (see Fig. 2 c, e, f, g, h).

Finally, in Table 2, it is clear that maxRD outperforms the full information case. The imprecision and persistence-distance values computed in maxRD samples are substantially lower than those obtained from the betweenness values of original graph *G*.

### Top-k nodes similarity

In the next paragraphs, we present the results of the OSim, with respect to degree centrality and k-core, obtained from maxRD samples.

The OSim is an object similarity measure, that is, the percentage of common nodes, between two ranking lists in a given top-k. As we mentioned in “Methods” section, the first ranking list is the ground truth ranking, while the second one is the ranking of nodes in a given sample, according to the centrality values that are computed in that sample/subgraph.

**Fig. 3:**
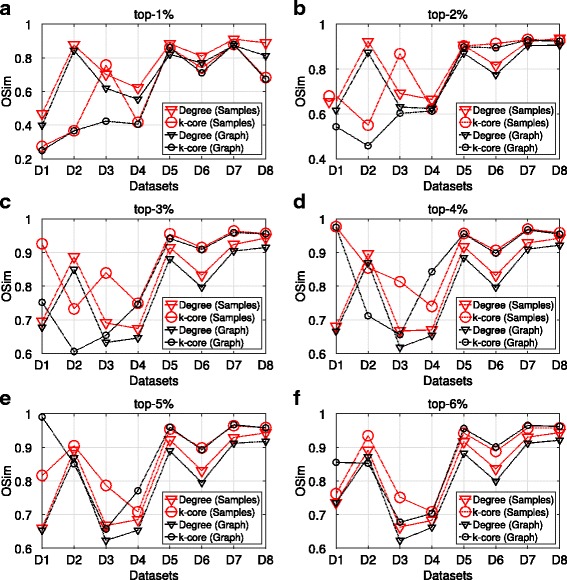
Top-k nodes similarity, SIR epidemic. **a**-**f** Average OSim values in terms of degree centrality and k-core obtained from the maxRD samples, as well as from the original graph

**Fig. 4:**
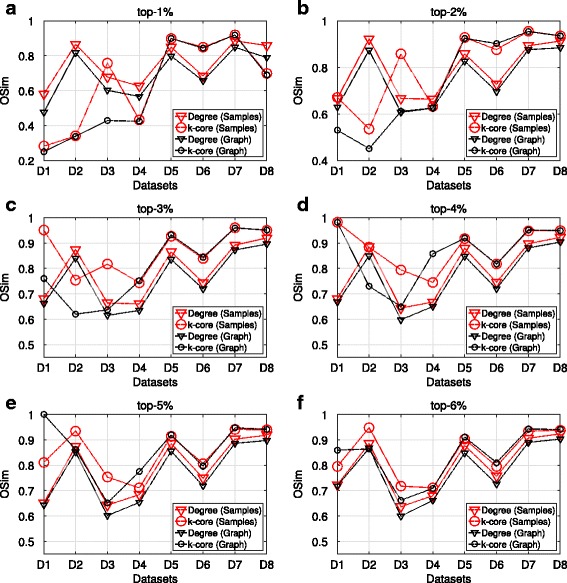
Top-k nodes similarity, SIS epidemic. **a**-**f** Average OSim values in terms of degree centrality and k-core obtained from the maxRD samples, as well as from the original graph

Figures 3 and 4 present the OSim values, in separate plots, for each top-k as well as for all datasets D1 to D8. For a given top-k, we plot the OSim values with reference to degree centrality and k-core, when the centrality measures have been computed in the maxRD samples/subgraphs, along with the OSim values that correspond to the original graph. In other words, we compare the accuracy of maxRD to the full information case.

In all datasets and in all top-k intervals, the accuracy of maxRD is equal or larger than the accuracy of the full information case. Generaly, the maxRD OSim values are larger than 0.6 for all datasets, except the D1 (*egoFacebook*) and top-1% (Fig. 3 a). Moreover, the OSim values are always larger than 0.8, in five out of eight datasets in SIR and six out of eight in SIS, for at least one of the two centrality measures.

For instance, from Figs. 3 a and 4 a, we observe that in the top-1%, a very narrow interval, the maxRD identifies more than the 80% of the influential spreaders in five datasets, D2, D5 to D8.

An important result is that the maxRD OSim values, in terms of degree centrality, are generally very close to the OSim values with respect to the k-core, obtained from the original graph. This means that by sampling the 20% of the graph and by using only local information/measure, that is the degree centrality in the generated samples, we can approximate the accuracy of k-core (global information) computed in the original graph. We note that the k-core is generally more accurate than the degree centrality (see Kitsak et al. 2010).

### Ranking similarity

We continue the analysis examining the Kendall tau rank correlation coefficient measure. For a given graph *G*, top-k and centrality measure, we apply the Kendall tau measure between the ranked centrality values of the nodes in the maxRD samples with the centrality values that those nodes have in any position of the ground truth ranking. We follow the same procedures for the top-k nodes of the full information case, that is, when we compute the centrality values in the original Graph.

The Kendall tau values are computed only for the degree centrality because each k-core value is assigned to a group of nodes, hence many ties may occur.

**Fig. 5:**
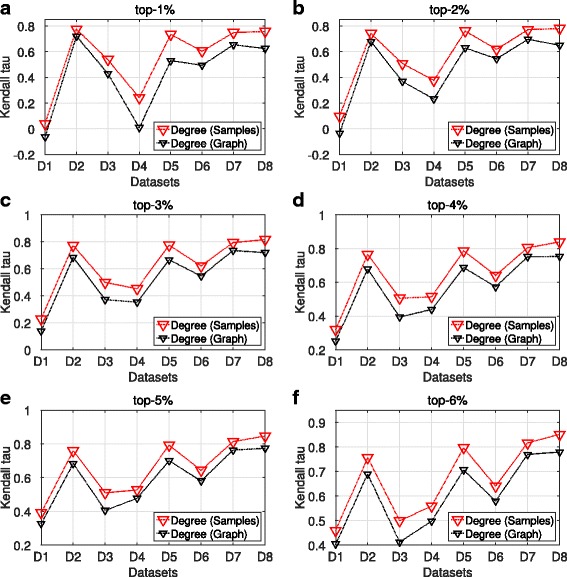
Ranking similarity, SIR epidemic. **a**-**f** Average Kendall tau values in terms of degree centrality obtained from maxRD samples, along with the Kendall tau values based on degree centrality obtained from the original graph

**Fig. 6:**
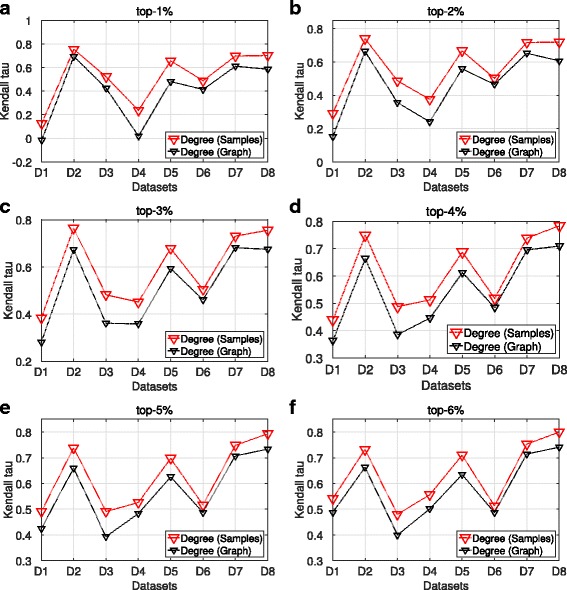
Ranking similarity, SIS epidemic. **a**-**f** Average Kendall tau values in terms of degree centrality obtained from maxRD samples, along with the Kendall tau values based on degree centrality obtained from the original graph

Figures 5 and 6 present the average Kendall tau values over the 100 maxRD samples, along with the Kendall tau values for the original graph, for each top-k separately.

We observe that in four out of eight datasets, (D2, D5, D7 and D8), the average Kendall tau values lie on the intervals (0.73,0.85) and (0.65,0.80) for SIR and SIS, respectively. Thus, there is a large positive correlation between the ordering of the top-k nodes in the samples and the ordering that these nodes have in the ground truth raniking. For instance, for every top-k, the Kendall value for the dataset D8 (*Slashdot0922*) - the largest of the eight datasets - is always very close to 0.8 in both epidemics. Moreover, maxRD outperforms the full information case in any dataset and top-k interval.

### Comparison of sampling methods

We continue the analysis, by comparing our method with three well known graph sampling algorithms: the Forest Fire (FF), the Metropolis Hastings Random Walk (MHRW) and the Metropolis Hastings (MH). We note that the MH is a “centralized” algorithm which takes as input the original graph; while running for thousands iterations, it performs a degree distribution approximation. For the sake of simplicity, only the results of SIR are shown. In SIS epidemic, the algorithms exhibit similar performance with the one in SIR.

**Fig. 7:**
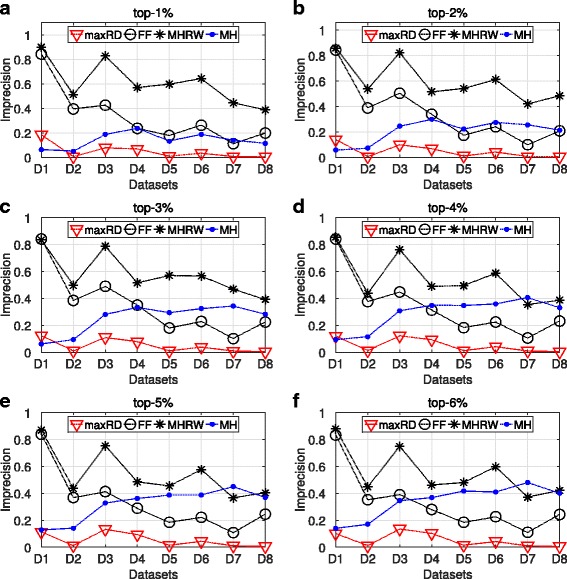
Comparison of the sampling methods. SIR epidemic. **a**-**f** average imprecision values based on the degree centrality values, obtained from the 100 samples of each method

**Fig. 8:**
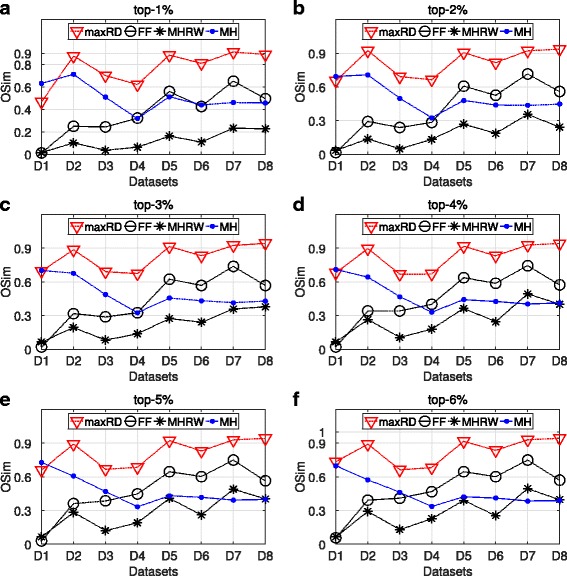
Comparison of the sampling methods. SIR epidemic. **a**-**f** average OSim values based on the degree centrality values, obtained from the 100 samples of each method

Figures 7 and 8 present the basic evaluation measure for the SIR epidemics, that is, the imprecision and OSim. We present the results for the degree centrality in separate plots, for each top-k interval. Let us note, that the imprecision and OSim values for the maxRD algorithm have already been presented in Figs. 1 and 3.

The maxRD significantly outperforms all the other three methods. Specifically, in four out of eight datasets (D2, D5, D7, D8), the maxRD imprecision values lie on the interval (0.0043,0.0140), for every top-k. On the other hand, the values of FF and MH are larger than 0.2, in at least half of the datasets (see Fig. 7). Only in the D1 dataset (*egoFacebook*), the MH exhibits similar performance with maxRD. The Metropolis Hastings Random Walk, in particular, is the one which presents the lowest performance.

As regards the nodes similarity in Fig. 8, the OSim values of FF and MH are generally significantly lower than those of maxRD. Finally, the performance of MHRW is very low, as the OSim values are always smaller than 0.3, in at least five out of eight datasets.

### Effect of the size of the initial seeds

We conclude the analysis investigating the relation between the effectiveness of maxRD and the size of initial seeds, that is the initial starting nodes of the algorithm (see Algorithm 1, Step 4). Thus, we compare the maxRD which starts from 1% of the graph to the extreme case where the algorithm starts only from one node. We note that the former case is the one that we analyzed in the previous sections. The latter case is in fact a parameter free exploration algorithm which performs only one graph traverse by visiting one node at each time step.

In Table 3, we present the OSim values for the SIR epidemics with reference to degree centrality and k-core, for both maxRD versions. Similar are the results for the SIS epidemic. For simplicity of the presentation we present only the SIR case.

**Table 3:** OSim values (SIR epidemic) in terms of degree centrality and k-core for two maxRD versions: (i) the number of initial seeds is equal to 1% and (ii) the number of initial seeds is equal to 1

		Top-1%		Top-2%		Top-3%		Top-4%		Top-5%	
	Seeds	1%	1	1%	1	1%	1	1%	1	1%	1
Degree	D1	0.4683	0.4740	0.6546	0.6596	0.6966	0.6993	0.6802	0.6843	0.6604	0.6699
	D2	0.8756	0.8775	0.9230	0.9210	0.8867	0.8874	0.8965	0.8961	0.8919	0.8921
	D3	0.7013	0.7019	0.6915	0.6878	0.6910	0.6920	0.6672	0.6661	0.6673	0.6724
	D4	0.6212	0.6147	0.6645	0.6599	0.6742	0.6754	0.6704	0.6666	0.6851	0.6827
	D5	0.8834	0.8786	0.9055	0.9019	0.9149	0.9150	0.9178	0.9156	0.9221	0.9202
	D6	0.8099	0.8071	0.8167	0.8179	0.8327	0.8334	0.8321	0.8339	0.8307	0.8330
	D7	0.9112	0.9100	0.9217	0.9201	0.9241	0.9229	0.9286	0.9275	0.9290	0.9282
	D8	0.8897	0.8878	0.9370	0.9357	0.9434	0.9417	0.9425	0.9413	0.9431	0.9425
k-core	D1	0.2732	0.2695	0.6799	0.6656	0.9259	0.9148	0.9768	0.9868	0.8158	0.8763
	D2	0.3665	0.3641	0.5501	0.5015	0.7325	0.6851	0.8543	0.7973	0.9049	0.8990
	D3	0.7565	0.7519	0.8684	0.8682	0.8400	0.8394	0.8139	0.8164	0.7869	0.8101
	D4	0.4175	0.4022	0.6232	0.6042	0.7491	0.7334	0.7398	0.7575	0.7081	0.7194
	D5	0.8604	0.8610	0.9026	0.9008	0.9554	0.9488	0.9565	0.9575	0.9537	0.9576
	D6	0.7397	0.7347	0.9135	0.9100	0.9144	0.9159	0.9062	0.9077	0.8977	0.8998
	D7	0.8801	0.8801	0.9318	0.9307	0.9642	0.9636	0.9700	0.9696	0.9639	0.9662
	D8	0.6826	0.6825	0.9269	0.9268	0.9565	0.9565	0.9587	0.9578	0.9585	0.9584

The results clearly indicate that both versions are almost equivalent. In every dataset and top-k, the OSim values for both versions are almost equal even for the k-core measure. This depicts an important result, that the size of the initial seeds does not affect the accuracy of the method. This fact can simplify further the design of the method and increase its usability in real world applications. The maxRD is an exploration algorithm easy to implement and can be used for crawling online social networks. In the past few years, the complex networks (especially the social ones) have considerably increased in size. This fact increases the importance of sampling in network analysis. Generally, the studies of the influential spreaders identification problem implicitly assume that the whole original network is available for investigation. In real world networks, this approach is not realistic, as the networks due to their large size are usually unknown.

## Conclusion

In this paper, we have presented a graph exploration sampling method that accurately identifies the influential spreaders in a complex network. The proposed method is based on a simplified version of *Rank Degree* graph sampling algorithm. We have performed an extensive experimental analysis on eight real world datasets of different types, using three centrality measures, as well as two well known epidemic models, which served as ground truth identifiers of nodes spreading efficiency. Moreover, we have compared our method with three well known sampling algorithms. The experimental analysis provides strong evidence for the effectiveness of the method which identifies the influential spreaders, with high accuracy, by sampling only 20% of the network.
